# Cognitive Bias Modification versus CBT in Reducing Adolescent Social Anxiety: A Randomized Controlled Trial

**DOI:** 10.1371/journal.pone.0064355

**Published:** 2013-05-14

**Authors:** B. Esther Sportel, Eva de Hullu, Peter J. de Jong, Maaike H. Nauta

**Affiliations:** 1 Department of Psychiatry, University Medical Center Groningen, University of Groningen, Groningen, The Netherlands; 2 Department of Clinical Psychology, University of Groningen, Groningen, The Netherlands; Catholic University of Sacred Heart of Rome, Italy

## Abstract

Social anxiety is a common mental disorder among adolescents and is associated with detrimental long term outcomes. Therefore, this study investigated the efficacy of two possible early interventions for adolescent social anxiety and test anxiety. An internet-based cognitive bias modification (CBM; n = 86) was compared to a school-based cognitive behavioral group training (CBT; n = 84) and a control group (n = 70) in reducing symptoms of social and test anxiety in high socially and/or test anxious adolescents aged 13–15 years. Participants (n = 240) were randomized at school level over the three conditions. CBM consisted of a 20-session at home internet-delivered training; CBT was a 10-session at school group training with homework assignments; the control group received no training. Participants were assessed before and after the intervention and at 6 and 12 month follow-up. At 6 month follow-up CBT resulted in lower social anxiety than the control condition, while for CBM, this effect was only trend-significant. At 12 month follow-up this initial benefit was no longer present. Test anxiety decreased more in the CBT condition relative to the control condition in both short and long term. Interestingly, in the long term, participants in the CBM condition improved more with regard to automatic threat-related associations than both other conditions. The results indicate that the interventions resulted in a faster decline of social anxiety symptoms, whereas the eventual end point of social anxiety was not affected. Test anxiety was influenced in the long term by the CBT intervention, and CBM lead to increased positive automatic threat-related associations.

**Trial Registration:**

TrialRegister.nl NTR965

## Introduction

Social anxiety disorder (SAD) is one of the most common mental disorders in children and adolescents, with about 9.5% of girls and 4.9% of boys facing social anxiety disorder in their adolescent period (14–24 years old [1]). Social anxiety is associated with poor development of social skills, reduced social interactions, low self esteem and lower academic performance [2], as well as future comorbid anxiety disorders, depression, and substance abuse [3]. Because of the pervasive impact of social anxiety disorder on current and future well-being, early detection and intervention of SAD seems of paramount importance. Previous research has shown that prevention and early intervention in a school setting can be effective in reducing anxiety symptoms and in preventing the onset of anxiety disorders in general, both at short and long term (see [4] for a review).

Current cognitive models emphasize the role of threat-confirming information processing biases in the development and maintenance of anxiety and imply a reciprocal relationship between fear and threat-confirming cognitive biases [5]. Socially anxious people are known to show an attentional bias towards threat [6] and to interpret ambiguous information in a relatively negative way [7]. These biased information processes are hypothesized to be an etiological and maintaining factor in anxiety and therefore could serve as a target for symptom reduction and early intervention.

Thus far, most interventions for social anxiety have focused on explicit, verbalizable cognitions, such as Cognitive Behavioral Therapy (CBT). This type of interventions has been shown to be effective in reducing anxiety symptoms [8], [9] and in preventing the onset of anxiety disorders in a school setting with effect sizes in the small to moderate range [4]. Recent research suggests that it might also be feasible to more directly target cognitive biases. There is accumulating evidence that (social) anxiety can be reduced through Cognitive Bias Modification (CBM) procedures focusing on interpretive bias [10] or attentional bias [11]. A central aim of this study was to test if CBM might also be efficacious in early intervention and symptom reduction.

Biased information processing is already involved in adolescents with a subclinical level of social anxiety. Recent studies have shown that high socially anxious adolescents, when compared to low-socially anxious adolescents, show relatively more negative automatic threat-related associations [12] and more negative and less positive interpretations of ambiguous social situations [13]. Both high and low socially anxious adolescents show an initial attentional bias towards threatening faces and words [13]. In the current study, we designed a Cognitive Bias Modification training to target attentional bias, interpretive bias, dysfunctional associations, and implicit self esteem in socially anxious adolescents. Based on the argument made by Hirsch, Clark, and Mathews [14] that cognitive biases are likely to be mutually reinforcing, we chose to include tasks that modify different biases into one training, thus allowing for effects of the training on interpretive bias to interact with effects on attentional bias and vice versa in an attempt to maximize the efficacy of the training. We combined some well established paradigms such as a word fragment task to modify interpretive bias [15] and a modified visual probe task to modify attentional bias [16] with less-often used paradigms, such as a conditioning paradigm to modify implicit social anxiety associations [17] and a classical conditioning task to amplify self-related positive associations [18].

The CBM intervention was contrasted with a more traditional CBT group training. In the present CBT-based intervention we integrated ingredients that have been shown to be effective in the treatment of social anxiety in children and adolescents. We created an intervention based on current golden-standard treatment protocols [19], [20], adjusted for the purpose of early intervention in a Dutch sample of adolescents. It has been shown that effective CBT studies in children and adolescents typically used cognitive restructuring and exposure techniques (for a review see [21]). In our effort to tailor the intervention to social anxiety, we added psycho-education based on the model by Clark and Wells [22]. In line with this model emphasizing self-awareness, we also included Task Concentration Training (TCT, [23]), which is recommended as a treatment for SAD in the Dutch clinical guidelines [24].

In short, Cognitive Bias Modification and Cognitive Behavioral Group training were contrasted with a no-treatment control condition. Both types of training were rolled out in a school-based setting and focused on adolescents (age 13–15) with mild to moderate symptoms of social anxiety. Since social anxiety in adolescents often takes the form of test anxiety (e.g., fear of poor performance on tests or in front of an audience [25]) and test anxiety is claimed to be of major concern for educational institutions [26], we also focused on this component of social anxiety in the content of the interventions and added test anxiety symptoms next to social anxiety as a primary outcome measure in the present design. Finally, since current dual process models emphasize the importance to differentiate between deliberate self-reports and more automatically activated associations [27], the efficacy of both interventions was not only indexed by structured interviews and self-report questionnaires but also by a performance measure of social anxiety-relevant automatic associations [12].

## Methods

### Design & Ethics Statement

The protocol for this trial and supporting CONSORT checklist are available as supporting information; see Checklist S1 and Protocol S1. The current project was conducted in collaboration with secondary schools, and was started in the context of a request from one of the schools for evidence-based interventions for youth with social and test anxiety. The study used a multi-arm parallel group approach and employed a stratified design with balanced randomization (1∶1∶1). It was approved by the medical ethics committee of the University Medical Center Groningen, the Netherlands. All participants, together with at least one parent or caretaker, provided written informed consent prior to the start of the study. The study was registered in the Dutch trial register with number NTR965 [28]. Power analysis showed that for a medium effect, with a power of .80, within three groups, with an alpha of .05 (one-sided), the sample size had to be 52 for each condition. Because of anticipated drop-out we aimed at 75 participants per condition. Recruitment took place in 2007 and 2008; all assessments took place between 2007 and 2011.

### Participants

**Figure 1:**
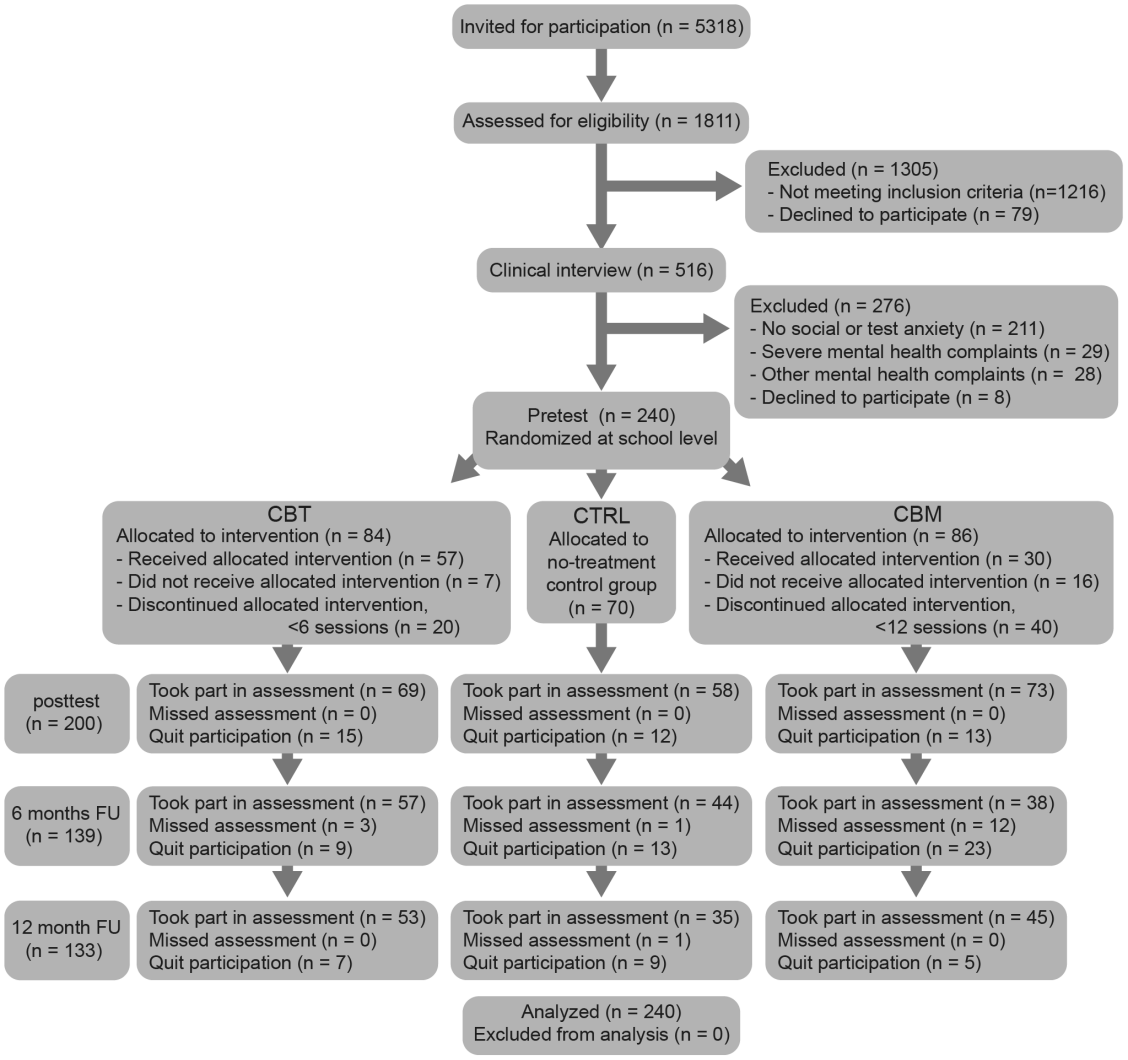
Study overview.

We invited 5318 adolescents in the first and second year of regular secondary schools in the Northern part of the Netherlands for the initial screening (see Figure 1 for flow diagram). Participants who handed in the required informed consent forms (N = 1811) were screened using the Revised Child Anxiety and Depression Scale (RCADS, [29]) and the Spielberger's Test Anxiety Inventory [30]. Participants scoring above cut-off for social and/or test anxiety (n = 516) were invited for a clinical assessment using the Anxiety Disorders Interview Schedule for Children (ADIS-C [31]). Used cut-off scores for girls were >10 on RCADS social phobia and >43 on TAI, cut-off scores for boys were >9 on RCADS social phobia and >38 on TAI. The RCADS cut-off scores were based on the 75th percentile in a large Dutch cohort of young adolescents (N = 2230, the TRAILS-study [32]), TAI cut-off scores were based on the 75th percentile in the Dutch manual [30]. Screening took place in two waves, including 12 schools in the first year and 13 schools in the second year.

Based on the ADIS-C, adolescents with low-level social anxiety were included in the current study (N = 240; age 12–15; 66 boys), Table 1 provides an overview of baseline participant characteristics for each condition.

**Table 1:** Baseline participant characteristics by condition (Cognitive Bias Modification/ Cognitive Behavioral Group Training /no-treatment control).

	CBM n = 86	CBT n = 84	Control n = 70	* F*	* χ^2^*	* p*
Gender (n (%) girls)	66 (77%)	56 (67%)	54 (77%)		2.94	.236
Age	14.12 (0.66)	14.06 (0.73)	14.11 (0.55)	0.23		.779
Social anxiety (RCADS)	13.64 (4.95)	13.11 (4.26)	13.27 (4.52)	0.30		.742
Test anxiety (STAI)	41.09 (13.94)	41.82 (13.28)	41.59 (13.23)	0.06		.938
stIAT	−0.02 (0.35)	−0.03 (0.29)	0.00 (0.27)	0.22		.802
ADIS-C (n (%) of participants)						
CSR = 4	14 (16.3%)	9 (10.7%)	8 (11.4%)		1.36	.544
CSR = 3	23 (26.7%)	15 (17.9%)	13 (18.6%)		2.43	.309
CSR = 2	9 (10.5%)	14 (16.7%)	9 (12.9%)		1.43	.491
CSR = 1	0 (0.0%)	2 (2.4%)	0 (0.0%)		3.75	.206
CSR = 0	40 (46.5%)	44 (52.4%)	40 (57.1%)		1.77	.413

* Note: Variables show mean (standard deviation) unless denoted otherwise. Participants with CSR  = 0 did not meet the full criteria for social anxiety disorder, these adolescents met at least DSM-IV criteria A and B for social anxiety disorder, showing fear of negative evaluation in multiple social contexts.

For ethical reasons, adolescents with DSM IV diagnoses other than anxiety and/or with severely interfering anxiety diagnoses and/or who expressed a need for regular treatment were referred to regular mental health centers to receive a regular evidence-based intervention. After the pretest, participants were randomized in a stratified design at school-level over one of three conditions (see Figure 1). Based on the number of participating adolescents, schools were grouped in three equally sized clusters of three schools. The three clusters of schools were randomly allocated to one of the three conditions. This procedure guaranteed that the number of participants would be similar across conditions. Of the 24 participating schools, 8 schools received CBT, 7 schools CBM, and 7 schools were assigned to the control condition. In two small schools no students were eligible for inclusion. The allocation of the schools was done by the project leader, by blindly drawing same size papers with the conditions CBT, CBM or Control from a bowl (in the presence of the last author). Neither participants, nor researchers supervising the assessments did receive information about the condition until after the pretest, to make sure condition was not of influence in the testing nor in the willingness to participate. Not all participants completed all assessments: post-test (n = 200), 6 months follow-up (n = 139), 12 months follow-up (n = 133). Drop-out did not differ between conditions (*Χ^2^* (6)  = 4.58, *p* = .60).

### Interventions

The CBM intervention consisted of 20 sessions (40 minutes each), delivered twice a week via the internet. Participants received information explaining the rationale of the training. Each week, participants received an e-mail with links to two training sessions (Table 2), and were reminded if they did not complete a session. The backbone of CBM consisted of tasks to modify interpretation (9 sessions) and attention bias (8 sessions). The *interpretive bias* (IB) modification tasks were constructed along the lines of the CBM-I designed by Mathews and Mackintosh [15]. Participants were presented with ambiguous social scenarios (60 trials/session) that were followed by word fragments that had to be solved in a benign direction. We added an imagination training to the first session and before each task; participants were instructed to visualize the scenarios, since previous research has shown that this may amplify the task's effectiveness [33].

**Table 2:** Order of tasks in the CBM training.

week	1	2	3	4	5	6	7	8	9	10
First task	IB	ABa	IB	AA	ABb	IB	IB	IB	AA	ABb
				SE	SE				SE	SE
Second task	IB	ABa	AA	IB	ABb	ABa	ABa	ABb	IB	IB
		SE	SE		SE	SE	SE	SE		

Note. IB  =  interpretive bias task; ABa  =  attentional bias task, stimulus disappears at probe onset; ABb  =  attentional bias task, stimulus remains on screen; AA  =  automatic association task; SE  =  implicit self esteem enhancement task.

The *attention bias* (AB) modification tasks (8 sessions of 450 trials) were based on the visual probe task and the exogenous cueing task (cf. [34]). The aim was to guide participants to point their initial attention (stimulus presentation time was 500 ms) at positive (happy faces/positive words) or neutral stimuli and away from threatening stimuli (faces or words expressing social rejection). Participants were instructed to indicate as fast as possible whether the small arrow (probe) that appeared 500 ms after stimulus onset was directed upwards or downwards. Presentation time of the probe was tailored to individual performance. If the probe was identified correctly for more than 75% of the trials, in the next block the presentation time of the probe arrow decreased with 25 ms, and in the same way it increased when performance was poor. This tailoring kept the task at the right level of difficulty for individual participants. For half of the sessions (4 out of 8 sessions), the stimulus did not disappear upon probe presentation but remained on the screen, allowing for prolonged attention to the benign stimulus. We also included two less established tasks. First, we added a task (3 sessions of 500 trials) that aimed to strengthen the association between social-evaluative situations and positive outcomes. Participants sorted words related to (social) evaluative situations (exam), neutral words (chair), and positive outcome words (success) into two categories: Dutch or English. Social cues and positive outcome words were both consistently presented in Dutch, and thus shared one response button. Second, a short evaluative conditioning task [17], [18] of 240 trials was added to 10 sessions, aiming to enhance implicit self-esteem by associating self-relevant information (e.g., name, first letter of name, hometown) with positive outcomes.

The CBT intervention consisted of ten weekly sessions of 1.5 hours that were delivered in small groups (3−10 participants) by a licensed (CBT) psychologist, at school, after school hours. Components were: 1. psycho-education, aiming at recognizing and understanding anxiety symptoms using the model of Clark and Wells [22] as the starting point (session 1, 2); 2. TCT (following [35]), to improve participants' awareness of their attentional focus, and to improve attentional control (session 3, 4); 3. cognitive restructuring, focusing on the identification / modification of dysfunctional thoughts (session 5, 6); 4. exposure, practicing with anxiety provoking situations (session 7, 8, 9). The last session (10) focused on how to avoid personal pitfalls and relapse. Participants also received homework assignments. The training protocol is highly structured and contains detailed information on all interventions, including some verbatim text fragments; the workbook includes background information and exercises for adolescents. Both materials can be received upon request.

Both interventions took approximately 1.5 hours a week, with a total duration of ten weeks.

### Control group

One cluster of participating schools was randomly allocated to the no-intervention control group. After the pretest, participants in these schools received a letter explaining that they formed the control group and thus were invited to all assessments but would not receive the PASTA training. It was stated that they were free to seek treatment if they felt the need, but none of the participants did actually seek treatment elsewhere during this study.

### Training attendance

On average, participants in the CBM condition completed 8.5 out of 20 CBM sessions (standard deviation (SD)  = 6.9) while participants in the CBT condition attended 6.7 sessions out of 10 CBT sessions (SD = 3.3). A proportion of participants in the CBM condition (*n* = 16) did not start the CBM training, mostly due to technical difficulties.

### Outcome measures

Social anxiety symptoms were indexed by the social phobia subscale (9 items) of the Revised Child Anxiety and Depression Scale (RCADS, [29]) with items rated on a 4-point scale ranging from 0 (never) to 3 (always). Internal consistency of the RCADS-SP was satisfactory (at pretest α = .79).

Test anxiety was indexed by the Spielberger Test Anxiety Inventory (Spielberger TAI, [30]), with 20 items rated on a 4-point scale, ranging from 1 (almost never) to 4 (all the time). In the current study, reliability at pretest proved to be excellent (α = .95).

As an implicit measure of social anxiety symptoms we assessed threat-related automatic associations by means of a Single Target Implicit Association Test (stIAT) with the target category ‘social or school activity’, and attribute labels positive versus negative outcome (see [12] for details). StIAT scores were computed according to the algorithm proposed by Greenwald [36], which recently has shown to perform also best in a laboratory setting [37]. In this paper, we report the so-called D4 measure, with a 600 ms error penalty for incorrect responses. A high score indicates relatively strong automatic associations between social or school activities and positive outcomes. Split-half reliability as indexed by Spearman-Brown corrected coefficient was .72 for the stIAT.

To assess the presence of SAD during pretest and posttest, we carried out clinical interviews using the anxiety and mood sections of the ADIS-C [31]. In the current sample, the interrater-reliability was very high with 99.7% overlap (based on ratings by a psychologist and independent rater scoring a random selection (n = 30) of the available ADIS-C interviews (n = 248) from pretest.

### Procedure

The assessments were performed on laptops at school, during or after school hours. Measures were presented in fixed order. After the pretest, participants were informed about the assigned condition. Posttest was after 12 weeks, followed by follow-up assessments at 6 and 12 months. Participants received a gift certificate (5 Euro) for each assessment. The ADIS-C at posttest was conducted via telephone. Interviewers remained blind for participants' condition.

### Change in cognitive biases

For assessment of the effect of the CBM training on participants' cognitive biases, participants completed several tasks before and after the training period. To examine attentional bias to social threat, we used two versions of a visual probe task that was specifically designed for this study: one using pictorial stimuli (Visual Probe task with Faces; VPF) and one using verbal stimuli (Visual Probe task with written Words; VPW). Each visual probe task comprised 76 trials; 12 practice trials (neutral-neutral, stimuli not present in the critical trials) and 64 critical trials (32 positive-neutral and 32 negative-neutral). Trials ran in a fixed random order. Stimuli were presented supraliminally on a white background. On each trial a black fixation cross appeared for 500 ms followed by a stimulus pair presented horizontally for 500 ms. Probes were small black arrows pointing upwards or downwards, presented immediately after the stimuli disappeared. In the VPF, stimuli were neutral, friendly (happy) and threatening (contempt) faces, selected from the Karolinska Directed Emotional Faces series (KDEF, [38]), showing straight profile images of 32 men and 32 women. Each stimulus pair consisted of two pictures of faces belonging to the same individual, either friendly-neutral or threatening-neutral. In the VPW, stimuli were 64 different word pairs, matched for number of characters (3−11), with fixed random presentation of 32 combinations of neutral (*spoon, curtain*) – friendly (*smile, success*) and 32 combinations of neutral (*stove, blanket*) – threatening (*shame, failure*) words.

To assess changes in interpretive bias, two tasks were used: the Recognition task and the Adolescent Interpretation and Belief Questionnaire (AIBQ, [39]). The Recognition Task was adapted from earlier versions [7], [15] such that the scenarios presented were appropriate for adolescents in a school environment. On the computer screen, participants read a scenario of a social situation, followed by a word fragment that they were asked to solve. The (social) situation remained ambiguous, and a comprehension question appeared which made sure that participants had read the text. Incorrect answers on the comprehension questions are an indicator that the participant did not read the scenario carefully, such that the answers to the recognition question will not reflect actual interpretations but guesses. After 10 trials describing various social situations, the title of the description was repeated and participants were asked to rate the similarity (1 =  *very similar in meaning* to 4 =  *very different in meaning*) of four different interpretations (positive, negative, neutral, or irrelevant) of the situation to the original situation that they have read before. Positive and negative interpretive biases are calculated from the ratings on positive and negative interpretations. Mean scores for the 10 situations are reversed such that higher scores indicate a higher (positive or negative) interpretive bias. The AIBQ is a questionnaire designed to assess interpretations and beliefs about both social and non-social ambiguous situations in adolescents. An example of an item measuring interpretive bias for social situations is as follows: *You*'*ve invited a group of classmates to your birthday party, but a few have not yet said if they*'*re coming. Why haven*'*t they said something yet?* After this description, three interpretations of the situation (positive, negative, and neutral) were presented individually and respondents were asked to rate how likely it is that this interpretation would pop up in their mind (1 =  *does not pop up in my mind* to 5 =  *definitely pops up in my mind*). Interpretive bias was calculated by adding up the scores from each interpretation/situation combination divided by the number of situations (5), resulting in a range with minimum 1 (no bias) to 5 (strong bias).

### Statistical Analyses

Multilevel analysis, using MLwiN Version 2.18 [40], was used to answer the research questions whether (a) cognitive biases did change as a result of cognitive bias modification, (b) the two training conditions were effective in reducing symptoms of social anxiety and (c) whether one of the training conditions was more effective than the other. Since missing data analysis indicated that data was missing at random (MAR), multilevel modeling provides an elegant method for dealing with missing data, taking all available data into account without the need for imputation [41], [42]. Multilevel models were estimated for the three outcome measures of social and test anxiety, namely RCADS Social Phobia, Spielberger TAI and stIAT. As a first step in the modeling, we defined the assessment session as a first level and participant as second level. School could have been added as a third level, however, exploratory analyses showed no effect of school. School was found to hold 0% up to 2.2% of the variance, and was therefore not included as a grouping variable in further analyses. Next, an unconditional model was employed to estimate the variance partitioned at each level. In a more specific model, looking into the various time segments, the categorical variable time (assessment point) was added, with random slopes for level 2. For the conditional model, third, the interaction variable time x training condition was added in a fixed manner, with control condition and pretest as reference categories. This model is also used for reporting change in cognitive biases between pretest and posttest, where we replaced the outcome variable (e.g., social anxiety) by the reported bias index (e.g., interpretive bias). Finally, an overall model was created by adding time and group x time interaction, with pretest and 12 month follow-up as markers, to get an idea of the overall change within and between the groups. We checked whether these models could be improved by including treatment attendance as covariate. The reported effect sizes for group differences are derived from the differences between groups at time points, reported effect sizes over time were derived from differences between time points. All analyses were conducted following the intent-to-treat principle, including all 240 participants.

For analyzing possible group differences at start, t-tests and Pearsons *Χ* ^2^ tests were used when comparing two means (e.g., for differences between completers and non-completers), and ANOVA or Pearsons *Χ* ^2^ test was used when comparing more than two means (e.g., for differences between the three conditions at start).

## Results

### Change in cognitive biases

Multilevel analysis, using MLwiN Version 2.18 [40] was used to answer the question whether information processing in the CBM condition developed differently from CBT and CTRL conditions between pretest and posttest. For interpretive bias as measured by the recognition task, interpretations became less negative in the CBM condition compared to both the control group (coefficient  = −0.48, SE  = 0.08, *p*<.001) and the CBT condition (coefficient  = −0.46, SE  = 0.08, *p*<.001. Interpretations became more positive in the CBM condition compared to both the control group (coefficient  = 0.43, SE  = 0.08, *p*<.001) and the CBT condition (coefficient  = 0.43, SE  = 0.08, *p*<.001). For social interpretive bias as measured by the AIBQ, interpretations became less negative in the CBM condition compared to the control group (coefficient  = −0.33, *SE*  = 0.14, *p* = .008). Positive social interpretations generally increased (time effect coefficient  = 0.33, *SE*  = 0.10, *p* = .001) but there was no effect of condition. For attentional bias to threatening faces, there were no significant effects of time or condition. Attentional bias to friendly faces increased in the CBM condition compared to the control group (coefficient  = 20.12, *SE*  = 9.53, *p* = .017). All in all, these results provide (at least partial) support for the efficacy of CBM to modify the targeted cognitive biases (see [13] for a thorough discussion of all process measures).

### Missing Data

A detailed overview of the participant flow is provided in Figure 1. In total, 33 of the 86 participants in CBM, 50 of the 84 participants in CBT, and 34 of the 70 participants in the control condition completed all four test sessions. There was no indication of selective attrition. That is, at pretest, there were no differences between participants who completed all test sessions and those who only completed pre-test (RCADS-sp: t = −0.55, p = .58; TAI: t = 0.48, p = .64; stIAT: t = −0.57, p = .57).

### Descriptive Statistics

Means and standard deviations for the outcome measures as a function of test session are shown in Table 3. At pretest, there were no differences between conditions (RCADS-sp: F(2,239) =  0.30, *p* = .74; TAI: *F*(2,120)  = 0.02, p = .99; stIAT: F(2,227) =  0.16, *p* = .85). For the ADIS-C diagnosis of Social Anxiety Disorder (SAD), we performed a Pearson's *Χ* ^2^ analysis using a dichotomic variable, which is 1 when SAD is present (in cases with a CSR of 4 or higher on the ADIS-C) or 0 in the absence of SAD. At pretest, no differences were found between conditions (*Χ^2^* (2)  = 1.36, *p* = .54). In the CBT condition, 9 out of 84 (10.7%) met criteria for SAD compared to 14 out of 86 (16.3%) in the CBM condition and 8 out of 70 (11.4%) in the CTRL condition.

**Table 3:** Means and standard deviations of RCADS, TAI and stIAT at the four assessment points by condition (Cognitive Bias Modification/ Cognitive Behavioral Group Training /no-treatment control).

	CBM		CBT		Control	
Dependent	Mean	SD	Mean	SD	Mean	SD
RCADS social phobia						
pretest	13.64	4.95	13.11	4.26	13.27	4.52
posttest	11.34	5.42	12.35	4.84	11.59	4.75
6 month FU	10.00	5.91	9.71	3.71	11.48	4.89
12 month FU	10.15	5.73	10.13	4.70	10.94	4.55
Spielberger TAI Test Anxiety						
pretest	41.09	13.94	41.82	13.28	41.59	13.23
posttest	35.51	11.47	34.76	10.82	38.56	13.17
6 month FU	34.27	12.09	31.11	8.63	37.36	12.44
12 month FU	32.62	11.83	31.58	9.67	35.14	11.08
stIAT Automatic Threat-related Associations						
pretest	−0.02	0.35	−0.03	0.29	0.00	0.27
posttest	−0.01	0.27	−0.11	0.29	−0.03	0.28
6 month FU	0.00	0.29	−0.08	0.34	−0.01	0.29
12 month FU	0.07	0.27	−0.10	0.29	−0.06	0.26

### Differences across conditions for the various time segments

To test the interventions' efficacy we subjected the three outcome measures to multilevel analysis.

**Figure 2:**
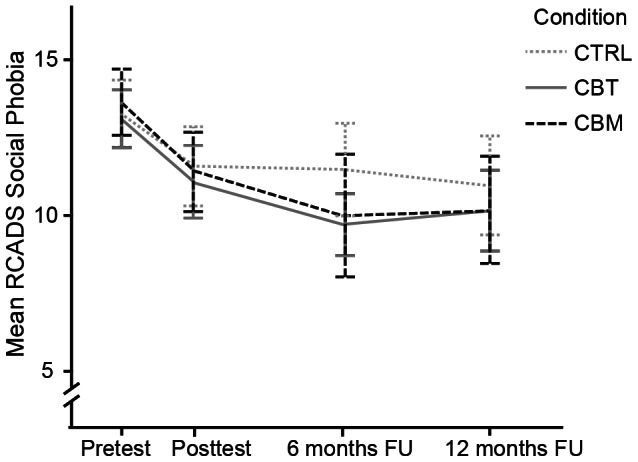
RCADS Social Phobia over time for Cognitive Bias Modification (CBM), Cognitive Behavioral Group Training (CBT) and no-treatment control group (CTRL).

As can be seen in Figure 2 and Table 4, already in the first segment there was an overall decrease in RCADS Social Phobia scores (ES: Cohen's *d* = 0.42). From posttest to 6 month FU the coefficients of the time x group interaction show that the subsequent reduction was stronger within the CBT condition than within the control condition (ES: Cohen's *d* = 0.41). For the CBM condition the pattern was similar, although the difference between CBM and the control condition did not reach significance (coefficient = −1.48, SE = 1.06, p = .08). A significant overall decrease in test anxiety (TAI scores) was found between pretest and posttest (ES: Cohen's *d* = 0.42). The CBT group showed a significantly stronger reduction of test anxiety scores compared to the control condition between pretest and posttest and from posttest to 6 months FU (ES: Cohen's *d* = 0.32 and *d* = 0.58 respectively). For the stIAT no overall time effects emerged. Yet, during the first segment there was a time x condition interaction indicating that CBT showed less reduction in negative associations than both the control (ES: Cohen's *d* = 0.28), and CBM condition (ES: Cohen's *d* = 0.36). For this segment, no differences were found between the control condition and CBM. From 6 to 12 months follow-up, the further increase in positive automatic associations was found to be stronger for CBM (ES: Cohen's *d* = 0.61) than for both the CBT and the control condition.

**Table 4:** Estimated effects for the conditional models between pretest – posttest, posttest – 6 months follow-up and 6 months follow-up –12 month follow-up for Cognitive Bias Modification (CBM) vs. Cognitive Behavioral Group Training (CBT) vs. No-treatment control group.

	RCADS Social Phobia		TAI Test Anxiety		stIAT Automatic Threat-related Associations	
	β	SE	β	SE	β	SE
Time effect						
Intercept	13.35	0.30	41.49	0.78	−0.02	0.02
Posttest vs pretest	−1.68**	0.73	−3.20*	1.80	0.00	0.04
6 mth FU vs posttest	0.14	0.79	1.30	1.89	0.04	0.05
12 mth FU vs 6 mth FU	0.09	0.89	1.18	2.09	−0.02	0.06
CBM vs control						
Posttest vs pretest	−0.27	0.89	−2.56	2.16	0.01	0.05
6 mth FU vs posttest	−1.48	1.06	−3.09	2.53	−0.01	0.07
12 mth FU vs 6 mth FU	−0.50	1.05	−2.53	2.48	0.13*	0.07
CBT vs control						
Posttest vs pretest	−0.58	0.90	−3.67*	2.18	−0.09*	0.05
6 mth FU vs posttest	−1.76*	0.96	−6.26***	2.28	−0.06	0.06
12 mth FU vs 6 mth FU	−0.40	1.03	−3.56	2.34	−0.04	0.06
CBM vs CBT						
Posttest vs pretest	0.32	0.84	1.11	2.03	0.10*	0.05
6 mth FU vs posttest	0.29	1.02	3.16	2.40	0.05	0.07
12 mth FU vs 6 mth FU	−0.10	0.95	1.04	2.21	0.17**	0.06

Note: * p<.05; ** p<.01, *** p<.001.

### Efficacy at one-year follow up

Table 5 provides an overview of the results for the long term efficacy of the interventions. Most critical, the RCADS scores decreased between pretest and 12 month follow-up (coefficient = −0.21, SE = 0.04, *p*<.001, ES: Cohen's *d* = .64); yet this effect was not especially pronounced for CBM/CBT conditions (*p*>.15). Test anxiety decreased between pretest and 12 month follow-up (coefficient = −0.56, SE = 0.09, *p*<.001, ES: Cohen's *d* = 0.71), with a significant overall difference between the CBT and the control condition (coefficient = −0.40, SE = 0.18, *p* = .01, ES: Cohen's *d* = 0.34). For the stIAT, there was no overall effect of time (coefficient = 0.00, SE<0.01, *p* = .50). However, there was a significant time x condition effect for CBM versus CBT (coefficient = 0.01, SE<0.01, *p* = .003, ES: Cohen's *d* = 0.61), with the CBM condition showing a stronger reduction in threat-related associations.

**Table 5:** Estimated effects for the conditional models between pretest and 12 months follow-up for Cognitive Bias Modification (CBM) vs Cognitive Behavioral Group Training (CBT) vs No-treatment control group (CTRL).

	RCADS Social Phobia		TAI Test Anxiety		stIAT Automatic Threat-related Associations	
	β	SE	β	SE	β	SE
Intercept	12.73	0.22	39.89	0.60	−0.03	0.02
Time effect	−0.21***	0.04	−0.56***	0.09	0.00	0.00
CBM vs CTRL	−0.06	0.08	−0.25	0.19	0.01	0.00
CBT vs CTRL	−0.08	0.08	−0.40*	0.18	−0.01	0.00
CBM vs CBT	0.03	0.08	0.15	0.17	0.01**	0.00

* *p*<.05; ** *p*<.01, *** *p*<.001.

### Presence of Social Anxiety (ADIS-C)

At posttest, the number of social anxiety disorder diagnoses in each group was 8 out of 68 (11.8%) in the CBT condition, 9 out of 68 (13.2%) in the CBM condition and 2 out of 57 (3.5%) in the no treatment control condition. Since only a small fraction of the participants received a diagnosis of SAD, these data could not meaningfully be subjected to statistical analysis to test change over time or differences between groups. These numbers differ slightly from the numbers in the flow chart; seven participants filled out the questionnaires, but did not participate in the ADIS-C interview.

### Influence of treatment attendance

For both conditions, the overall number of attended sessions was not related to the level of social anxiety at posttest (coefficient = −0.01, SE = 0.01, *p* = .22). Yet, there was an effect for pretreatment social anxiety indicating that individuals with lower initial anxiety attended fewer sessions (coefficient = 0.19, SE = 0.07, *p* = .003). On average, participants in the CBT condition attended 6.7 sessions (out of 10 sessions; SD = 3.3) and in the CBM condition 8.5 sessions (out of 20 sessions, SD = 6.9). The mean (standard deviation in parentheses) number of tasks completed in CBM was 5.00 (2.57) for interpretive bias; 3.71 (2.75) for attentional bias; 2.13 (0.80) for automatic associations and 5.61 (3.08) for self-esteem tasks.

## Discussion

### Summary of main findings

This study was the first to test the efficacy of CBM in an early intervention study, using a multifaceted CBM approach. The major findings can be summarized as follows: (i) In the short run (6 months follow-up) participants in the CBT condition showed a larger reduction in social anxiety symptoms than participants in the control condition, and a similar trend was evident in the CBM condition, with effect sizes in the small to moderate range, (ii) In the long run (12 months follow-up) the control condition eventually showed a similar reduction in social anxiety symptoms as both active conditions, (iii) After CBT, adolescents reported a stronger decrease of test anxiety compared to the no-intervention control group, (iv) From post-test to 12 months follow up the CBM group showed a stronger decrease of negative automatic associations than both the CBT and the no-intervention control group.

### Effects of CBM and CBT on social anxiety

Regarding our main explicit outcome measure for social anxiety (Revised Child Anxiety and Depression Scale, social phobia subscale [29]) we found an overall improvement over time. In addition, we found a relatively strong improvement in the CBT condition at 6 months follow-up, and a similar trend for CBM. An advantage for the active conditions was not evident immediately following the intervention (i.e., at posttest). In prevention research (see [4] for a review) it is common that effects are not visible directly after the intervention, which may also count for our participants with relatively low levels of social anxiety. In the present study, this lack of effect may at least partly be due to the fact that our social anxiety questionnaire did not give a specific instruction on reporting on the recent weeks. Participants may have reported on their behavior in general over the last months, thus reducing the sensitivity of this instrument to detect immediate improvement. Moreover, it seems reasonable to assume that after training, participants still need further practice and reassuring experiences in concrete social situations before they actually correct their original (dysfunctional) cognitions. The difference between the active conditions and control condition at 6 month follow-up may thus be regarded as the actual treatment effect: participants had time to practice the newly learned skills and/or to experience the corrective impact of the interventions on habitual information processing strategies. This finding is comparable to Aune and Stiles [43], who tested the efficacy of a universal CBT program and found a prevention effect for syndromal and subsyndromal social anxiety 8 months after the active intervention period.

At 12 month follow-up, we found no differences between the three conditions in social anxiety. Participants in the control condition further improved, whereas participants in both training conditions remained at the same level of social anxiety. One explanation could be that participants in the training condition had already approached normal levels of social anxiety at post treatment. In line with this, Chorpita et al. [29] reported an average of 11.7–12.3 on the RCADS social phobia scale in this age group in a normal sample, where our post-intervention scores at 12 month follow-up were between 10.1 and 10.9. However, direct comparison of our scores to a Dutch population sample seem to indicate that the scores in our sample were still above the normal level at post-treatment (>1 standard deviation of the mean score), and only within the normal range at 12 months follow-up [44]. Thus, it seems that there was still sufficient room for further improvement. Perhaps it could be beneficial in this respect to add booster training sessions during the follow up period. This may not only help to further decrease the level of symptoms and to prevent the recurrence of symptoms but may also stimulate/motivate the participants to further train their acquired skills. It would be important for future research to examine whether indeed this type of additional components would help to further improve these interventions.

### Effect of CBM and CBT on test anxiety

Over time, CBT did result in a stronger decrease of test anxiety than the no-intervention control condition. This decrease in test anxiety may well reflect a direct effect of the CBT group training, since specifically in this condition, participants learn to actively cope with their test anxiety. In the CBM condition, quite some scenario's in the interpretive bias task focus on test-anxiety specific situations, but participants received no help to directly cope with acute test anxiety.

### Effects of CBM and CBT on clinical diagnoses of Social Anxiety Disorder

In the present study we not only examined the impact of the interventions on the level of self-reported social anxiety, but also investigated whether the interventions would be effective in preventing the development of social anxiety disorder (SAD). The results of the ADIS-C diagnostic interview showed that, overall, the number of diagnoses of SAD was very low in all groups including the no-intervention control group. The absence of a substantial number of SAD diagnoses rendered it impossible to test the efficacy of our interventions to prevent the development of SAD. It remains therefore to be tested (e.g., on the basis of a longer follow up period) whether the present intervention can also be used to actually prevent the development of SAD.

### Automatic evaluative threat-related associations

Interestingly, CBM showed a more favorable effect in reducing automatic social threat-related associations than CBT in all time segments. Specifically in the longer-term (from 6 to 12 months FU), the CBM condition also showed a more favorable effect than the controls. Although within the present time frame CBM did not have a more favorable effect on self reported social anxiety than the control condition, it would be interesting to see whether in the longer term differential effects may arise. Moreover, it would be interesting to add social tasks to the verbal assessments, since the reduction of automatic associations through CBM might be especially effective in modifying relatively spontaneous fear behaviors [27]. Together, the pattern of findings with regard to participantś automatic threat associations not only supports the efficacy of CBM, but also points to the relevance of complementing the routinely used self-report measures with performance based measures that may be more sensitive to automatically activated associations in memory.

### CBM Treatment Integrity

Since attentional bias to threat was hypothesized to be an important factor in adolescent social anxiety, we added a large number of attentional bias training sessions to the multifaceted CBM training in the current study. We expected attentional bias to change in the CBM condition into a more benign pattern of attention to friendly stimuli and attention away from threat. These expectations, however, were not confirmed. Although attentional bias to friendly faces did change in the short term, this change was very small and we did not find a similar change in attentional bias to friendly words. The effects of CBM on interpretive bias were more convincing. In the CBM condition, interpretive bias measured using the recognition task became more positive and less negative during the training period. Earlier research [7], [45] showed that changes in interpretive bias caused by CBM seldom generalize to other measures of interpretive bias, but we found that interpretative bias as measured by the AIBQ changed as well; negative interpretive bias decreased in the CBM condition relative to the control condition and all groups developed a more positive interpretation style. All in all, the present findings provided partial support for the efficacy of CBM to modify the targeted processes, thereby confirming its validity; especially as a method to modify interpretive bias.

The CBM combined multiple tasks in an attempt to increase its efficacy. Yet, studies in CBM with favorable effects have thus far focused on single cognitive mechanisms. Thus, it can not be ruled out that the combination of tasks might in fact have led to suboptimal effects. The finding that the decrease in automatic threat-associations was most pronounced for the CBM condition nevertheless supports the validity of the CBM approach. Future research is required to test which element of our CBM was most effective in decreasing associations to threat. In our CBM, only 3 sessions were directly devoted to modifying automatic associations. Perhaps increasing the number of these sessions could improve the efficacy of CBM in reducing the strength of automatic threat associations. In addition, it is worth noting that CBM did not result in a convincing reduction of attentional bias, whereas interpretive bias was strongly reduced. This suggests that the impact of the present CBM approach might improve further by focusing more on interpretive bias and/or by attempts to improve the efficacy of the attentional bias tasks.

### Methodological considerations and limitations of the study

Some comments are in order regarding the limitations of the current study. First, training attendance was quite low, especially in the CBM condition, which may have been a factor in the generally small effects of the interventions. Nevertheless, in other CBM studies with favorable effects on anxiety, the entire CBM program usually contains fewer sessions (e.g., 8 sessions of attentional bias modification [34]) and the number of attended treatment sessions in our training appeared to be unrelated to the later level of social anxiety. This latter, counterintuitive, finding may be explained by the relationship between number of attended training sessions and the initial level of social anxiety. Highly anxious participants completed more sessions than those with less anxiety. Probably, motivation to continue the training was lower in participants with less anxiety who were also more assertive in declaring that they wanted to quit, thereby increasing the chances of drop-out. Training attendance could probably be improved by limiting technical difficulties in the CBM condition (e.g., an operating system/browser-independent CBM training that does not need separate plug-ins to be installed) and offering (financial) incentives to complete CBM (or CBT) sessions. Second, although the finding that the decrease in automatic threat-associations was most pronounced for the CBM condition is promising and supports the validity of the CBM approach, future research is required to test which element of the CBM training is most effective in decreasing associations to threat. Furthermore, earlier research has demonstrated that automatic associations may be especially relevant in guiding more spontaneous fear behaviors [46]. Unfortunately, the present study did not include indices of relatively spontaneous fear behaviors (e.g., heart rate during an actual evaluative conversation). For a more comprehensive appreciation of the relevance of the relatively strong reduction of the automatic associations in the CBM condition, it would be important for future research to include such tasks as an additional outcome measure.

Finally, it should be acknowledged that we received informed consent from only one-third of the invited adolescents and their parents. Therefore, we cannot rule out the influence of selection bias on the present findings. The medical ethics committee did not allow further contact with the non-responders, leaving the reasons for their non-response unclear. In the information letter, the aim of the study was pointed out, which may have led to non-response in a particular subsample of anxious adolescents. In addition, despite reminders about upcoming assessments through telephone and e-mail, financial incentives for attended assessments, and rescheduled missed assessments, a considerable number of participants dropped out during various stages of the project. We believe that this high drop-out rate reflects the reality of at-school intervention research and could not be prevented. Fortunately, drop-outs did not differ on important variables such as age, gender, and initial anxiety levels from those who completed all assessments, thus justifying our use of multilevel analyses. However, with a small effect size to be expected in a sample of moderately anxious adolescents, the limited power remains problematic. Definitive conclusions on the efficacy of early interventions such as described here should be drawn on the basis of meta-analysis of multiple studies [47].

### Conclusions

In sum, the current study showed that our early CBT intervention has a beneficial effect in terms of reducing test anxiety. In the mid long term (6 months follow-up) this early CBT intervention also resulted in a relatively strong decrease in social anxiety with a similar trend for CBM. However, in the longer term (12 months follow-up) this training benefit disappeared. Importantly, the automatic social threat-related associations weakened most following CBM (specifically in the longer term). This seems especially relevant in light of earlier findings showing that this type of automatic associations have prognostic value for the future onset and unfavorable course of anxiety disorders [48], [49]. It would be important for future research to test the relative efficacy of the various components of the early CBM intervention. On the basis of such findings the optimal combination of effective components could be selected, which in turn might help to improve further the efficacy of CBM as a tool to prevent the generation and/or persistence of SAD symptoms.

## Supporting Information

Checklist S1 **CONSORT Checklist.**
(DOC)

Protocol S1 **Trial Protocol.**
(DOC)
