# Supplementary material for: Cognitive Bias Modification versus CBT in Reducing Adolescent Social Anxiety: A Randomized Controlled Trial
Source: PLoS One. 2013 May 14;8(5):e64355. doi: 10.1371/journal.pone.0064355 (PMC3653919; doi:10.1371/journal.pone.0064355)
Supplement: Protocol S1 — Trial Protocol. (DOC) [file pone.0064355.s002.doc]

**Onderzoeksprotocol**

**Preventie van psychische klachten bij middelbare scholieren**

Accare Groningen ^1^

Klinische & Ontwikkelingspsychologie RuG ^2^

Vakgroep Psychiatrie UMCG ^3^

Groningen, versie 14 februari 2006

Auteurs: Maaike Nauta ^1,2^, Peter de Jong ^2^, Ruud Minderaa ^1,3^

**Angst voor negatieve beoordeling bij faalangst en sociale angst bij middelbare scholieren: interventies ter preventie van psychische klachten**

| Protocol ID | 2006.005 |
| --- | --- |
| Verkorte titel | Preventie van psychische klachten bij middelbare scholieren |
| Versie | 2 |
| Datum | 14februari 2006 |
| Projectleider | *Dr. M.H. Nauta*  *Accare, UCKJP Groningen*  *Postbus 660*  *9700 AR Groningen* |
| Hoofdonderzoekers | *Dr. M.H. Nauta*  *Prof. dr. P.J. de Jong, Klinische & Ontwikkelings Psychologie RuG*  *Prof. dr. Minderaa, disciplinegroep Psychiatrie UMCG* |
| Projectleden | *Dr. P.P. Mersch, disciplinegroep Psychiatrie UMCG*  *Prof. dr. J.A. den Boer, disciplinegroep Psychiatrie UMCG*  *Drs. J.H. Loonstra, orthopedagoog Steunpunt Menso Altingh college* |
| Sponsor | *ZonMW Preventie*  *Contactpersoon: Margreet Bloemers*  *Postbus 93245*  *2509 AE Den Haag* |
| Onafhankelijk arts | *Dr. C. Reichart*  *Accare, UCKJP Groningen*  *Postbus 660*  *9700 AR Groningen* |

**PROTOCOL SIGNATURE SHEET**

| Naam | Handtekening | Datum |
| --- | --- | --- |
| Afdelingshoofd (bij niet-commerciele projecten):  *Prof.dr. Minderaa, hoogleraar en lid Raad van Bestuur Accare* |  |  |
| Projectleider / coördinerend onderzoeker:  dr. M.H. Nauta, GZ-psycholoog |  |  |

**INHOUDSOPGAVE**

SAMENVATTING 5

SUMMARY IN ENGLISH 6

1. INLEIDING EN RATIONALE 7

2. DOELEN 9

3. DESIGN VAN HET ONDERZOEK 10

4. ONDERZOEKSPOPULATIE 13

4.1. Populatie 13

4.2. Inclusie-criteria 13

4.3. Exclusie-criteria 13

4.4. Berekening steekproefgrootte 13

5. INTERVENTIES 14

6. METHODE 16

6.1. Procedure 16

6.1.1. Eerste fase: screening 16
6.1.2. Tweede fase: diagnostische verdieping 16

6.2. Meetinstrumenten 16

6.2.1. Primaire (expliciete) maten 16

6.2.2. Secundaire (expliciete) uitkomstmaten 17
6.2.3. Secundaire (impliciete) uitkomstmaten: 17

7. STATISTISCHE ANALYSES 19

8. ETHISCHE OVERWEGINGEN EN JURIDISCHE ASPECTEN 20

8.1. Goed Gedrag 20

8.2. Werving en toestemming 20

8.3. Verwachte baten en kosten voor deelname aan de studie 20

8.4. Informatieverstrekking aan patiënten over een ongunstig beloop 20

8.5. beloning voor deelname 20

9. ADMINISTRATIE EN DATAVERWERKING 21

9.1. Databeheer 21

9.2. Informatie over de resultaten van de studie 21

**SAMENVATTING**

Bij zowel faalangst als sociale angst staat de angst voor negatieve beoordeling centraal. Dit soort zorgen ontwikkelen zich vaak gedurende de puberteit en wanneer een puber erg veel van dit soort angst kent kan dit een behoorlijke impact hebben op het huidig en later functioneren. Jongeren die echt last hebben van deze angsten functioneren sociaal vaak minder goed, hebben minder goede sociale vaardigheden, hebben een lagere zelfwaardering, hebben lagere cijfers en lopen een hoger risico om de school vroegtijdig zonder diploma te verlaten. Op langere termijn kan sociale angst leiden tot ernstige klachten zoals depressie of alcoholmisbruik.

Slechts een kleine minderheid van de jongeren met ernstige sociale angst (zo'n 20%) vindt de weg naar de juiste hulpverlening.

Het huidige project onderzoekt de effectiviteit van een laagdrempelige groepsinterventie op middelbare scholen ter preventie van sociale fobie en gerelateerde psychische klachten. De effectiviteit van deze interventie zal worden vergeleken met de effecten van een nieuw ontwikkelde computergestuurde training die zich richt op het veranderen van informatieverwerkingsprocessen, en met een controlegroep zonder interventie.

2306 middelbare scholieren (13-15 jaar) worden gescreend op angst voor negatieve beoordeling met behulp van vragenlijsten. Van deze 2306 kinderen wordt 15% (n = 346) uitgenodigd voor verdere diagnostiek. Jongeren die al ernstige problematiek hebben, worden rechtstreeks verwezen naar Accare (polikliniek kinder- en jeugdpsychiatrie). Vervolgens zullen leerlingen uit de studie uitvallen omdat ze te weinig klachten hebben of niet willen of kunnen deelnemen aan de groepen. Uiteindelijk worden 225 kinderen gerandomiseerd over de drie behandelcondities. Er vinden bij deze 225 leerlingen vijf metingen plaats, namelijk voor behandeling, na behandeling, en na 6, 12 en 24 maanden. De meting na 24 maanden vindt plaats bij alle 2306 scholieren, om te onderzoeken of de kinderen die oorspronkelijk als `niet at-risk' werden gekenmerkt ook daadwerkelijk geen klachten ontwikkelden. Primaire uitkomstmaten zijn symptomen van sociale angst en faalangst, schoolcijfers en schoolverzuim. Secundaire maten zijn symptomen van depressie en alcoholmisbruik, en daarnaast impliciete angstmaten.

De belasting voor de grote groep leerlingen (screening) is twee maal 1 lesuur, met een tussenpoos van 2 jaar. In dat lesuur worden vragenlijsten ingevuld en computertaakjes uitgevoerd. De groep die deelneemt aan het interventie-onderdeel van de studie heeft 5 extra metingen, verspreid over 2 jaar, waaronder een aantal extra vragenlijsten en een klinisch interview. Voor zover ons bekend zijn er geen risico’s aan deelname aan het onderzoek verbonden.

**SUMMARY IN ENGLISH**

Fear of negative evaluation is the core concern in both test anxiety and social anxiety. These anxious concerns typically develop during adolescence and elevated levels of these concerns have a large impact on current and future functioning. Adolescents suffering from these concerns often have lower levels of social functioning, lower self esteem, lower marks at school, poorer social skills, and are at risk for dropping out of secondary school. Longitudinal studies have shown that social phobia is strongly linked with consequent severe psychological complaints such as depression and alcohol abuse. The detrimental influence of social phobic concerns on individuals' current and future functioning is further strenghtened by the fact that only a small minority of adolescents reaches professional care.
The current project tests the effectivity of easily accessible cognitive behavioural intervention groups at secondary schools for preventing the development of social phobia and related complaints. This intervention will be compared with the effects of both a recently developed computerised intervention program aiming at changing fear confirming information processing biases, and a no-intervention control group.
2306 secondary school children (aged 13-15 years) will be screened for fear of negative evaluation. Of these 2306 scholars, 225 who are `at risk' for developing social phobia will be included and randomised across the three conditions. In order to test short and long term effectiveness, five assessments are scheduled: pre, post, and at 6-, 12, and 24-months follow-up. Importantly, all 2306 scholars are reexamined at 24-months follow-up, to check whether this non-at-risk group indeed did not develop anxiety disorders. Primary outcome measures include symptoms of social anxiety and test anxiety, school grades, and school attendance. Secondary measures include symptoms of depression and substance misuse, as well as implicit measures of individuals' representations of the self (implicit self esteem) and of fear-relevant social situations.

**1. INLEIDING EN RATIONALE**

Bij sociale fobie en faalangst staat de angst voor afwijzing centraal. Bij jongeren is deze angst voor afwijzing verhoogd aanwezig (Gullone en King, 1993). Volwassenen met sociale fobie rapporteren dat de angsten begonnen in de puberteit. Sociale fobie komt veel voor bij jongeren met een lifetime prevalentie van 9.5% bij meisjes en 4.9% bij jongens (14-24 jaar; Wittchen et al., 1999). Sociale fobie komt veel voor en heeft grote gevolgen voor het functioneren van de jongere: ze hebben vaak minder vriend(inn)en, lagere zelfwaardering, minder goede sociale vaardigheden, slechtere schoolprestaties en ze lopen een verhoogde kans om de middelbare school zonder diploma te verlaten (Nauta, 2005). Daarnaast zijn er sterke aanwijzingen dat sociale fobie kan leiden tot andere klachten zoals depressie (31% van de jongeren), middelenmisbruik (41%) of andere angststoornissen (50%; zie Wittchen et al., 1999). In 80% van de gevallen van depressie, middelenmisbruik of angststoornissen was er sprake van sociale fobie in het jaar voorafgaand aan de start van de stoornis. Ondanks deze grote impact op het huidige en latere functioneren van de jongeren weet slechts een klein deel van de jongeren daadwerkelijk de weg naar de hulpverlening te vinden (21.5%).

De grote impact van sociale fobie op huidig en later functioneren (met alle maatschappelijke kosten van dien) maakt het aannemelijk om jongeren op te sporen die een verhoogde kans hebben op het ontwikkelen van sociale angsten en hen te onderwerpen aan interventies die in het verleden effectief bleken tegen sociale fobie. Deze interventies dienen laagdrempelig te zijn en via school te worden aangeboden.

Cognitieve gedragstherapie (CGT) is bewezen effectief voor jongeren met angststoornissen in het algemeen (Nauta et al., 2003) en scholieren met faalangst in het bijzonder (Wessel en Mersch, 1994). CGT is ook succesvol toegepast in preventieprogramma’s: zes maanden na een preventiestudie kelderde de incidentie van angststoornissen van 54% in de controlegroep naar 16% in de CGT-conditie (Dadds et al., 1997). Hoewel er dus enig onderzoek is geweest naar preventie, missen er toch belangrijke onderdelen:

1. Preventiestudies bij kinderen of jongeren warden voornamelijk uitgevoerd in de VS en Australië (Durlak en Wells, 1998). Het is onduidelijk of deze resultaten zomaar zijn te vertalen naar de Nederlandse situatie.

2. Op basis van de onderzoeksgegevens die er nu zijn, kunnen geen harde conclusies getrokken worden over de daadwerkelijke effectiviteit van CGT in het voorkómen van sociale fobie, depressie of middelenmisbruik, aangezien de methodologie niet voorzag in het beantwoorden van deze vraag. Om deze vraag goed te kunnen beantwoorden, zullen de interventie en geen-interventiegroepen beide moeten worden vervolgd voor de periode waarin zich normaliter de klachten ontwikkelen. Tevens is het belangrijk niet alleen de at-risk-leerlingen te evalueren, maar ook de andere groep leerlingen, om te kijken of de oorspronkelijke selectie van `at risk’ leerlingen wel terecht was, en in deze groep niet allerlei psychopathologie is ontstaan.

3. Tot nu toe vonden de preventiestudies meestal plaats op basisscholen, terwijl het begin van sociale fobie veel later is, namelijk in de puberteit. Het zou zo kunnen zijn dat het beter is om de jongeren juist in de puberteit de interventie aan te bieden, ten eerste omdat ze dan gemotiveerder zullen zijn om er zelf mee aan de slag te gaan, en ten tweede omdat de vaardigheden die ze op de basisschool leren niet zonder meer toepasbaar zijn op de middelbare school, in een andere ontwikkelingsfase met complexer interacties, verwachtingen en eisen.

4. Tot slot hebben de studies zich wat betreft uitkomstmaten tot nu toe vooral gericht op zelfrapportage en klinische interviews. Recent onderzoek wijst echter uit dat verandering in deze expliciete maten niet hoeft te betekenen dat er ook veranderingen in de disfunctionele automatische associaties (impliciet nivo) plaatsvinden (zie bijv., Teachman et al., 2003). De overgebleven disfunctionele associaties zouden wel eens een belangrijke rol kunnen spelen bij de terugkeer van de klachten (Wilson et al., 2000). Wellicht geeft de focus op de expliciete maten wel een vertekend, te positief beeld van de verandering door de interventies. Een combinatie van zowel expliciete als impliciete maten zal een genuanceerder beeld kunnen brengen.

De huidige studie heeft in de opzet rekening gehouden met al deze lacunes en hoopt aldus een helder antwoord te kunnen gaan geven op de vraag of training van middelbare scholieren met symptomen van faalangst en sociale angst leidt tot vermindering van de klachten en tot voorkoming van sociale fobie, depressie of middelenmisbruik.

**2. DOELEN**

Het hoofddoel van deze studie is om te toetsen of laagdrempelige groepstrainingen op middelbare scholen de ontwikkeling van sociale fobie en gerelateerde psychische problemen zoals depressie en alcoholmisbruik kunnen stoppen en/of voorkomen.

Het gaat hierbij om de volgende vragen:

1. Is cognitieve gedragstherapie (CGT) effectiever dan geen interventie in het verminderen of voorkomen van sociale angst en gerelateerde psychische problemen zoals depressie en alcoholmisbruik?

2. Is een computergestuurde training gericht op het veranderen van informatieverwerkingsprocessen (biasretraining, BRT) effectiever dan geen interventie en even effectief of effectiever dan CGT in het verminderen of voorkomen van sociale angst en gerelateerde psychische problemen zoals depressie en alcoholmisbruik?

Tot slot (3) zullen factoren worden opgespoord die de effectiviteit van CGT of BRT versnellen of ondermijnen of die voorspellend zijn voor terugval na behandeling. Het gaat hierbij om expliciete maten zoals demografische variabelen, sociale vaardigheden, temperament of gedragsproblemen en impliciete maten.

**3. DESIGN VAN HET ONDERZOEK**

In dit onderzoek gaat het om een geïndiceerd preventie programma: jongeren die risico lopen op het ontwikkelen van sociale fobie (vanwege hoge scores op angst voor beoordeling) worden opgespoord en behandeld. Het onderzoek is verder opgezet als een gerandomiseerde gecontroleerde studie (randomised controlled trial, RCT). De randomisatie vindt plaats op basis van schoolklassen jongeren en niet op basis van individuen (`randomised block design’). Belangrijkste reden hiervoor is om de condities zo zuiver mogelijk te houden. Wanneer jongeren uit dezelfde klas wel of geen actieve training krijgen zal dit ruis geven: jongeren kunnen elkaar materiaal laten zien of elkaar betrekken bij oefeningen.

Klassikale screening na toestemmingsverklaring van ouder en jongere

(n=2306, 13-15 jaar)

T0

Uitgebreidere diagnostiek bij 15% hoogscoorders en op verzoek van jongeren zelf (n=346)

T1

225 jongeren geschikt voor en akkoord met deelname groepstrainingen

121 jongeren zijn niet geschikt (te veel of te weinig klachten) of gaan niet akkoord

randomisatie

75 jongeren CGT

wekelijks 2 uur

12 weken lang

75 jongeren geen interventie

75 jongeren BRT

twee keer per week 20/30 min

12 weken lang

nameting (na 12 weken actieve of controle conditie) bij 225 jongeren

T2:

T3:

follow-up na 6 maanden bij 225 jongeren

T4:

follow-up na 12 maanden bij 225 jongeren

T5:

follow-up na 24 maanden bij 2306 jongeren

De totale projectduur van de studie is 5 jaar. De inclusie van scholieren is verdeeld over 2 jaar. In het schooljaar 2006/2007 zullen scholen deelnemen (vanaf hier scholen A genoemd) en in het jaar 2007/2008 zullen andere (locaties van) scholen deelnemen (B). Deze getrapte instroom in het project geeft meer garanties voor het daadwerkelijk includeren van het hoge benodigde aantal scholieren.

Het tijdspad is als volgt:
Maart - Juni 2006

- contacten met scholen
- definitieve afspraken maken op scholen (A)
- logistiek op scholen regelen (A)
- informatiebijeenkomsten organiseren voor ouders en leerkrachten (A)
- testbatterij verder ontwikkelen
- CGT draaiboek aanpassen
- Computerinterventie aanpassen voor deze doelgroep

September - december 2006

- Informed consent en eerste screeningen op scholen A (vragenlijsten en computertaken)
- Data-analyse eerste screening (A)
- Uitgebreidere diagnostiek van hoogscoorders (A)
- Randomisatie (A)
- Training van therapeuten

Januari - april 2007

- Interventies (scholen A)
- contacten met scholen (B)
- definitieve afspraken maken op scholen (B)
- logistiek op scholen regelen (B)
- informatiebijeenkomsten organiseren voor ouders en leerkrachten (B)

April 2007

- Nameting van deelnemers (3 condities; A)

September 2007

- follow-up 6 maand (3 condities; A)
- eerste screening op scholen (B)
- Data-analyse eerste screening (B)
- Uitgebreidere diagnostiek van hoogscoorders (B)
- Randomisatie (B)

Januari - april 2008

- Interventies (scholen B)

april 2008

- follow-up 12 maand (3 condities; A)
- nameting (3 condities; B)

september 2008

- follow-up 6 maand (3 condities; B)

april 2009

- tweede screening van alle deelnemers van de eerste screening (A)
- 12 maand follow-up (3 condities; B)

april 2010

- tweede screening van alle deelnemers van de eerste screening (B)

april 2005 - april 2010

- data-analyse en verslaglegging
- verslaglegging voor deelnemende ouders en jongeren
- verslaglegging in het Nederlands voor beleidsmakers, scholen, psychologen, etc.
- indien de actieve condities effectiever waren dan geen interventie: het beschikbaar maken van de interventies voor gebruik op scholen of in de hulpverlening

**4. ONDERZOEKSPOPULATIE**

**4.1. Populatie**

De 2306 deelnemende jongeren zijn 13-15-jarige leerlingen van de 2^e^ en 3^e^ klas van het middelbaar onderwijs. De scholen zullen zo representatief mogelijk zijn (verschillende schooltypes (VMBO/ HAVO/ VWO); stad en platteland; bijzonder en openbaar onderwijs). De 15% hoogstscorenden (n=346) worden uitgenodigd voor nadere diagnostiek. Jongeren met reeds ontwikkelde ernstige psychische klachten wordt aangeraden elders hulp te zoeken. De inschatting is dat ongeveer 225 jongeren zullen willen deelnemen aan het onderzoek.

**4.2. Inclusie-criteria**

Jongeren die op basis van de SCAS of ETAV een hoge score hebben en worden uitgenodigd voor verdere diagnostiek, en jongeren die zelf hebben aangegeven graag mee te willen doen aan de training.

**4.3. Exclusiecriteria**

Jongeren die een psychische stoornis (sociale fobie, gegeneraliseerde angststoornis of depressie) hebben en hier duidelijk belemmering van ondervinden kunnen niet deelnemen aan het onderzoek en worden verwezen voor verder diagnostiek en hulpverlening. Verder doen jongeren niet mee wanneer ze niet mee willen doen of niet mee kunnen doen vanwege praktische of organisatorische problemen.

**4.4. Berekening steekproefgrootte**

Om een significant verschil tussen 2 groepen te vinden met een medium effect size (bijvoorbeeld tussen CGT en controles of tussen BRT en controles) met een alfa van 0.05 dient iedere groep te bestaan uit tenminste 64 proefpersonen (Power analyse; Cohen, 1992). Gezien de mogelijke drop-outs van de studie is ervoor gekozen de 3 groepen (CGT, BRT, controles) ieder uit 75 leerlingen te laten bestaan. Deze 225 leerlingen komen uit een groep van 2306 middelbare scholieren. Van de 2306 leerlingen wordt de 15% hoogstscorende leerlingen uitgenodigd voor verdere diagnostiek (n=346). De veronderstelling is dat zo’n 10% van hen reeds aan een ernstige vorm van sociale fobie, gegeneraliseerde angststoornis of depressie zal lijden. Deze kinderen worden desgewenst rechtstreeks verwezen naar de polikliniek K&J psychiatrie van Accare voor verdere diagnostiek en reguliere behandeling en kunnen niet deelnemen aan de preventieve interventiegroepen in het kader van deze studie. 25% van de 346 hoogstscorende kinderen nemen waarschijnlijk niet deel aan de studie te weinig motivatie, andere duidelijke problemen, weigering, of problemen van praktische / organisatorische aard (n=86). 346 – 35 (te zware problemen) – 86 (willen niet deelnemen) = 225 kinderen ter randomisatie over de 3 groepen.

**5. INTERVENTIES**

De interventies zullen trainingen genoemd worden en geen therapie of behandeling, zodat de groepen laagdrempelig zijn voor de scholieren. De groepen zullen na schooltijd plaatsvinden, zoveel mogelijk op een rustige locatie binnen school.

1. **Cognitieve gedragstherapie (CGT):** cognitieve gedragstherapie is momenteel de behandeling van eerste keus voor angststoornissen (Durlak en Wells, 1998). Bij cognitieve gedragstherapie worden jongeren aangemoedigd om anders naar hun angstige gedachten te kijken en om situaties die ze lastig vinden in oplopende moeilijkheidsgraad op te zoeken. De training die in dit kader wordt gegeven is een aangepaste versie van de training van Wessel en Mersch (1994) en beslaat 12 sessies van 2 uur. De training bevat de volgende onderdelen: psycho-educatie, een CGT-model van sociale angst, het opsporen en uitdagen van gedachten rond prestatie en beoordeling en exposure-oefeningen.
2. **Bias ReTraining (BRT):** de nieuw ontwikkelde BRT (zie bijv. Mathews en MacLeod, 2002; Amir, 2004) lijkt in eerste onderzoeken bij volwassenen zeker zo effectief in CGT in het verminderen van sociale angst klachten (Amir et al., 2004; zie ook Dandeneau en Baldwin, in druk). De training bestaat uit computertaken die erop gericht zijn om functionele impliciete automatische informatieverwerkingsmechanismen te trainen. Functionele strategieën worden aangeleerd en bekrachtigd en disfunctionele strategieën worden verminderd. Bij het eerste onderdeel van BRT worden cursisten getraind om ambigue sociale informatie eerder als positief of optimistisch te interpreteren dan als negatief en pessimistisch (zie bijv. Mathews en MacLeod, 2002). Het tweede onderdeel van BRT stimuleert cursisten meer op positieve sociale informatie te letten dan op signalen van afkeuring en negatieve beoordeling zoals zij gewoonlijk doen (zie bijv. Amir et al., 2004). Gedurende het **eerste onderdeel** van de training verschijnen voor de deelnemer verhaaltjes van ambigue sociale situaties op het computerscherm waarbij van een aantal woorden slechts een paar letters staan vermeld. Het is de taak van de persoon om zo snel mogelijk de ontbrekende letters in te vullen en zo te komen tot een woord dat goed past in het verhaal. Een voorbeeld van zo'n situatie is: “Het is jouw beurt om een spreekbeurt te houden. Je staat voor de klas en begint met je verhaal. Op een gegeven moment hoor je rechts vooraan wat gefluister. Je kijkt naar de klasgenoten die daar rechts vooraan zitten. Ze kijken g...t.r…e..d.” . De letters zijn steeds zo gekozen dat er slechts woorden kunnen worden gevormd met een positieve of neutrale betekenis maar nooit de negatieve woorden die aansluiten bij de ongunstige (disfunctionele) interpretatie die sociaal angstige personen als regel maken in dit soort situaties (zoals ‘verveeld’). Omdat het hun taak is zo snel mogelijk de onvolledige woorden aan te vullen, wordt het maken van de habituele negatieve interpretaties van ambigue sociale situaties ontmoedigd, en het maken van functionele interpretaties juist bekrachtigd. De verwachting is dat de jongeren aldus hun gewoonlijke (disfunctionele) neiging afleren om sociale situaties als bedreigend te interpreteren (zie bijv Mathews en Macleod, 2002). Gedurende 6 weken krijgen de jongeren 2 sessies van 30 minuten per week (100 trials). Het **tweede onderdeel** van de BRT is een aandachtstraining, waarbij jongeren telkens op een computerscherm worden geconfronteerd met twee gezichten: een afwijzend / walgend en een vriendelijk / accepterend. Eén van de gezichten wordt achtereenvolgens vervangen door een letter (E of F), die na korte aanbieding ook weer verdwijnt van het scherm. De jongere dient zo snel mogelijk aan te geven of de letter een E of een F was. Meestal zal de letter verschijnen na het accepterende gezicht. Hierdoor leren de jongeren om hun aandacht eerder te richten op het accepterende dan op het afwijzende gezicht, aangezien die strategie de taak vergemakkelijkt. De deelnemende jongeren zullen aan 2 sessies van 20 minuten per week deelnemen (160 trials) (bijv., Amir et al., 2004).

**6. METHODE**

**6.1. Procedure**

De procedure valt uiteen in een procedure voor de screening van de grootste groep kinderen (n=2306) en een procedure voor deelnemers aan het onderzoek naar de training (n=225). In deze paragraaf worden afkortingen gebruikt die in de erop volgende paragraaf 6.2. worden toegelicht.

**6.1.1. Eerste fase: screening**De klassikale screening zal worden gedaan met behulp van de SCAS (Spence Children’s Anxiety Scale; Spence, 1998 en de ETAV (Examen en Toets Attitude Vragenlijst; van der Ploeg, 1984). Tevens voeren alle kinderen een aantal computertaken uit. De vragenlijsten en taken worden klassikaal op laptops afgenomen. 15% hoogst scorende kinderen worden uitgenodigd voor verdere diagnostiek (n=346). Tevens kunnen kinderen zelf aangeven graag in aanmerking te komen voor de verdere diagnostiek en de trainingen.

**6.1.2. Tweede fase: diagnostische verdieping**

Bij de 225 kinderen worden als extra diagnostiek de volgende maten afgenomen: onderdelen Sociale fobie, Gegeneraliseerde angststoornis en Depressie van een semi-gestructureerde interview (ADIS ouder en kindversie: Silverman en Nelles, 1988) en een gedragsvragenlijst (ouder- en kindversie; SDQ; Goodman, 1997). In het kader van het onderzoek worden in deze fase tevens maten van sociale vaardigheden (kind zelfrapportage MESSY; Matson, Rotatori & Helsen, 1983), cognities rond afwijzing (kind zelfrapportage FNE; Watson en Friend, 1979), temperament (ouderrapportage EATQ; Capaldi & Rothbard, 1992) en zorgconsumptie afgenomen.

- 1. **Meetinstrumenten**
     1. **Primaire (expliciete) maten**

*De Spence Child Anxiety Scale* (SCAS; Spence 1998; Nauta et al., submitted) is een zelfrapportagelijst voor kinderen en jongeren van 8-18 jaar en bestaat uit 38 items die symptomen van angst beschrijven. Items worden ingevuld op een 0 (nooit) tot 3 (altijd) schaal. De subschalen volgen de classificaties van de DSM-IV, namelijk sociale fobie, separatieangst, paniek / agorafobie, gegeneraliseerde angststoornis, dwang en angst voor lichamelijke verwonding. De vragenlijst is ontwikkeld in Australië en tevens uitgebreid onderzocht in Nederland (zie bijvoorbeeld Nauta, 2005; Muris, Schmidt en Merckelbach, 2000). De factor structuur bleek invariant over Australië en Nederland en subschaalscores waren vergelijkbaar. De voor deze studie relevante subschaal Sociale Fobie en de Totaalscore bleken goed te discimineren tussen kinderen met angststoornissen en controle-kinderen.

*De Examen / Toets Attitude Vragenlijst (ETAV; van der Ploeg, 1984)* is een veelgebruikte Nederlandse zelfrapportagelijst voor symptomen van faalangst. De lijst bestaat uit 20 items; de 2 schalen zijn Emotionaliteit en Piekeren.
*Het Anxiety Disorder Interview Schedule* (ADIS C/P: Silverman en Nelles, 1988) is een semi-gestructureerd interview en wordt afgenomen ten einde DSM-IV diagnoses te kunnen stellen. In het kader van de studie worden de onderdelen Sociale fobie, Gegeneraliseerde angst, Depressie en Middelenmisbruik afgenomen. Er is een kinder- en een ouderinterview, die beide worden afgenomen. Het interview met de ouders wordt telefonisch afgenomen. Duur van afname van de onderdelen wordt geschat op 45 minuten per interview.

*De Spence Child Anxiety Scale - parent version* (SCAS-p; Nauta et al., 2004) is vergelijkbaar met de SCAS, maar hier betreft het ouderrapportage.

**6.2.2. Secundaire (expliciete) uitkomstmaten***Het Anxiety Disorder Interview Schedule* (ADIS C/P: Silverman en Nelles, 1988) is een semi-gestructureerd interview en wordt afgenomen ten einde DSM-IV diagnoses te kunnen stellen. In het kader van de studie worden de onderdelen Sociale fobie, Gegeneraliseerde angst, Depressie en Middelenmisbruik afgenomen. Er is een kinder- en een ouderinterview, die beide worden afgenomen. Het interview met de ouders wordt telefonisch afgenomen. Duur van afname van de onderdelen wordt geschat op 45 minuten per interview.

*De Spence Child Anxiety Scale - parent version* (SCAS-p; Nauta et al., 2004) is vergelijkbaar met de SCAS, maar hier betreft het ouderrapportage.

- Fear of Negative Evaluation (FNE; Watson en Friend, 1979): een maat voor cognities rond sociale evaluatie, 30 items, wordt afgenomen ter beschrijving van de effecten van de behandelingen

- Strengths and Difficulties Questionnaire (SDQ; Goodman, 1997): brengt gedrags- en emotionele problemen in kaart; zowel ouder- als kinderrapportage, 25 items, wordt afgenomen om de groep als geheel in kaart te brengen (algemene psychopathologie)
- screeningsvragen rond middelenmisbruik; nader te bepalen in samenwerking met Trimbos-instituut. Zelfrapportage, max 20 items, wordt afgenomen als secundaire uitkomstmaat: het al dan niet ontwikkelen van middelenmisbruik

- Matson Evaluation of Social Skills with Youngsters (MESSY; Matson, Rotatori & Helsen, 1983): zelfrapportage, meet sociale vaardigheden, 62 items, wordt afgenomen ter beschrijving van de effecten van de behandelingen

- Early Adolescent Temperament Questionnaire (EATQ; Capaldi & Rothbard, 1992), ouderrapportage, 46 items, 6 subschalen, wordt afgenomen als mogelijke voorspeller van behandelresultaat op korte en langere termijn

- zorgconsumptie (in het kader van kostenefficiëntie). 1 keer per maand wordt de gerandomiseerde jongeren gevraagd of ze contacten hebben gehad met hulpverleners zoals huisarts, GGZ-medewerkers, mentoren, etc.
- de *rapportcijfers* van de 225 kinderen worden opgevraagd bij school voor alle rapporten tussen de eerste en de laatste meting. Tevens worden van hen gegevens over *Schoolverzuim* door school doorgegeven.

**6.2.3. Secundaire (impliciete) uitkomstmaten:**

De zogenaamde single target Implicit Association Test (stIAT; Greenwald et al., 1998; Huijding, 2006) zal worden gebruikt om eigenwaarde (self esteem) op een impliciete manier vast te stellen (zie de Jong, 2002) en om te meten in welke mate sociale situaties (zoals praatje, gesprek, afspraakje, beurt, spreekbeurt) zijn geassocieerd met positieve sociale uitkomsten (zoals compliment, gezelligheid) dan wel met negatieve uitkomsten zoals afwijzing en kritiek (zie de Jong et al., 2001). De IAT is in essentie een sorteertaak waarbij mensen de woorden die op het scherm verschijnen zo snel mogelijk moeten categoriseren door op de linker of de rechter knop te drukken. In de eigenwaarde stIAT is er een categorie Ik en een categorie Positief versus Negatief. In sommige fasen van de test moeten mensen met dezelfde knop reageren op woorden die passen bij Ik of Positief (bv. mij, ik, zelf; mooi, plezier, gelukkig) terwijl in andere fasen juist met dezelfde knop moet worden gereageerd op woorden die passen bij Ik of Negatief (bv. mij, ik, zelf; naar, gemeen, saai). De test is gebaseerd op het principe dat mensen relatief snel zijn als woorden die men bijelkaar vindt passen, de zelfde responsknop delen. Dus mensen met een positieve eigenwaarde zullen juist snel zijn in de fase dat Ik en Positief de responsknop delen en langzaam wanneer Ik en Negatief de knop delen, terwijl precies het omgekeerde geldt voor mensen met een negatieve eigenwaarde. Het verschil in reactiesnelheid tussen de Ik/Positief en Ik/Negatief fasen kan aldus worden gebruikt als een maat voor impliciete eigenwaarde. Op analoge manier wordt in een andere IAT het verschil in reactietijd vastgesteld tussen de fasen waarin sociale situatie woorden de knop delen met negatieve uitkomst woorden en fasen waarin sociale situatie woorden de knop delen met de positieve uitkomst woorden. Eerder onderzoek heeft laten zien dat dit type taak sensitief is voor interindividuele verschillen en kan onderscheiden tussen hoog en laagangstige personen (bv. de Jong, 2002; Huijding, 2006).

**7. STATISTISCHE ANALYSES**

Ter beantwoording van de eerste vraagstelling (het verschil in effectiviteit tussen de drie condities) zullen de uitkomstmaten worden onderworpen aan multilevel analyse. In multilevel analyse kunnen de variabelen worden genest in verschillende `levels’ (kenmerken die groepen gemeenschappelijk hebben), zoals school, trainingsgroep of therapeut. Deze analyses zijn geschikt aangezien de data afhankelijk zijn. Binnen deze analyses zal wat betreft missende data gewerkt worden met een `intent-to-treat-analysis’, waarbij data van uitvallers wel worden meegenomen in nametingen of follow-ups.
Residual Gain Scores (RGSs) zullen worden berekend om de voorspellende waarde van primaire of secundaire uitkomstmaten te berekenen. RGSs zijn de scores op de nameting (of f-up) minus de scores op de voormeting, vermenigvuldigd met de standaarddeviatie tussen beide. RGSs zijn een maat voor verbetering, waarbij wordt gecorrigeerd voor meetfouten. RGSs worden gecorreleerd met primaire en secundaire uitkomstmaten, om te analyseren of behandelresultaat dan wel de ontwikkeling van klachten mogelijk kan worden voorspeld op basis van de eerste screening (dan wel de voormeting).

**8. ETHISCHE OVERWEGINGEN EN JURIDISCHE ASPECTEN**

In dit hoofdstuk worden een aantal relevante ethische issues onder de loep genomen en uitgewerkt.

**8.1. Goed Gedrag**

Onderzoekers, interviewers en andere medewerkers van het onderzoek zullen zich houden aan de gedragscode gezondheidsonderzoek `Goed Gedrag’.

**8.2. Werving en toestemming**

De werving van scholen is reeds begonnen. Een grote middelbare school met verschillende onderwijstypes en locaties heeft reeds aangegeven grote belangstelling te hebben voor deelname (Menso Altingh, 3500 leerlingen); een orthopedagoog van het Steunpunt van de school zit in de projectgroep, en in het verleden is samenwerking geweest rond het organiseren van een symposium over `verborgen problematiek’ in het middelbaar onderwijs en het schrijven van een boek over dit thema (Loonstra en van der Vegt, 2005).

Andere scholen zullen nog benaderd worden, waarbij gezocht wordt naar een goede representativiteit (stad en platteland, VMBO / HAVO / VWO, bijzonder en openbaar onderwijs). Bijlage 1 betreft de brief aan de directie van scholen.

Wanneer de directie van een school akkoord is met deelname aan het project worden ook de leerkrachten en met name de mentoren van de klassen 2 en 3 geïnformeerd. Op scholen wordt desgewenst een informatieavond voor ouders en leerkrachten georganiseerd. Vervolgens worden tijdens een mentor-uur de informatiebrieven en toestemmingsformulieren voor leerling en ouders/ verzorgers (bijlage 2 en 3) uitgedeeld. Er volgt een bedenktijd van 1 week. Hierna worden hoogstscoorders uitgenodigd voor verdere diagnostiek, hetgeen wordt teruggekoppeld en waarna wordt gevraagd of de kinderen daadwerkelijk willen deelnemen aan een groepstraining. Na randomisatie worden leerlingen geïnformeerd over de loting door middel van een brief (bijlage 4). In de informatie staat dat men voor informatie over het onderzoek terecht kan bij de projectleider (M.H. Nauta) en voor inlichtingen en advies bij de onafhankelijk arts, mw. dr. Reichart, kinder- en jeugdpsychiater.

Kinderen en ouders kunnen zich te allen tijde terugtrekken uit het onderzoek zonder opgaaf van reden. Ze kunnen desgewenst de training blijven volgen.

**8.3. Verwachte baten en kosten voor deelname aan de studie**

De studie is opgezet als een preventie-studie. Op dit moment is onbekend of de genoemde trainingen effectiever zijn dan natuurlijk beloop in het voorkomen van psychische klachten. We weten wel dat de interventies baat hebben voor kinderen met daadwerkelijke ernstige psychische problemen (angststoornis of depressie), maar deze jongeren worden uitgesloten van deelname aan het onderzoek. Het gaat er juist om te onderzoeken of zo’n training ook een preventief effect zou kunnen hebben. Niet interveniëren zou wel eens even heilzaam of misschien te prefereren kunnen zijn.

**8.4. Informatieverstrekking aan patiënten over een ongunstig beloop**

Alle jongeren die deelnemen aan de screening (n=2306) krijgen de informatie uit de vragenlijsten direct teruggekoppeld na het invullen ervan (computer). Bij een kleinere groep (n=346) wordt tevens een ADIS afgenomen. De informatie uit de ADIS betreft een diagnose van sociale fobie, gegeneraliseerde angststoornis of depressie op basis van het verhaal van ouders of kind. Deze informatie is van het belang voor de gezondheid van het kind. Deze informatie wordt allereerst met het kind doorgesproken en daarna met de ouders. Een diagnose met een belemmering groter dan 5 op de ADIS op de eerste meting leidt tot uitsluiting van deelname aan het onderzoek. Ouders en kind worden geïnformeerd over de diagnose en mogelijkheden voor hulp. Zij worden daarbij geadviseerd contact op te nemen met de huisarts voor verwijzing naar Accare of een instelling voor GGZ. Bij Accare zijn mogelijkheden van snelle verwijzing voor diagnostiek en reguliere behandeling vanuit het onderzoek. Deze procedure wordt zowel bij de eerste ADIS gevolgd als bij de nameting of bij follow-ups.

**8.5. beloning voor deelname**

Er is voor gekozen om onder de jongeren die de vragenlijsten willen invullen en computertaken willen doen per 20 deelnemende leerlingen een CD-bon van 20 euro te verloten.

8.6. Verzekering

Nu het onderzoek naar zijn aard geen risico kent, heeft de oordelende commissie, de METc UMCG, ontheffing van de verzekeringsplicht verleend zoals bedoeld in art.4 lid 1 van het Besluit verplichte verzekering bij medisch-wetenschappelijk onderzoek met mensen.

**9. ADMINISTRATIE EN DATAVERWERKING**

**9.1. Databeheer**

Alle deelnemende leerlingen krijgen een onderzoeksnummer dat los staat van hun geboortedatum of initialen. Gezien de longitudinale opzet van de studie is het niet mogelijk om alles anoniem te houden; de gegevens van de eerste meting moeten immers aan de gegevens van de tweede meting gekoppeld kunnen worden. Er komt één lijst met onderzoeksnummers en persoonsgegevens zoals naam, school, klas en geboortedatum (`subject identification list’). Deze lijst is alleen ter inzage door de 2 (nog aan te stellen) AIO’s en wordt in een beveiligde file bewaard. Alle andere data-files met bijvoorbeeld de vragenlijst- en interviewgegevens zullen op onderzoeksnummer worden ingevoerd en geen namen of initialen bevatten. De `subject identification list’ blijft tot vijf jaar na afloop van het project bewaard en wordt daarna vernietigd.

**9.2. Informatie over de resultaten van de studie**

Aan het eind van de studie zullen de volgende mensen en organisaties worden geïnformeerd:

- de participerende jongeren en hun ouders
- de participerende scholen
- afdelingen Preventie van de GGZ
- Landelijk Kenniscentrum Kinder- en Jeugdpsychiatrie
- Medisch Ethische Toetsingscommissie UMCG
- ZONMW preventie
- Beleidsmakers relevante overheden
- Beroepsverenigingen zoals de VGCT, NVO, NIP, VKJP
- Patiëntenvereniging Angst, fobieën en dwang

Verder zullen de resultaten worden gepresenteerd op nationale en internationale congressen, en ter publicatie worden aangeboden in Nederlandstalige en internationale vaktijdschriften.

Wanneer één van de actieve condities aanmerkelijk betere resultaten geeft dan de controlegroep zal de projectgroep samen met deelnemende scholen een plan maken voor implementatie.
